# InsuOnline, an Electronic Game for Medical Education on Insulin Therapy: A Randomized Controlled Trial With Primary Care Physicians

**DOI:** 10.2196/jmir.6944

**Published:** 2017-03-09

**Authors:** Leandro Arthur Diehl, Rodrigo Martins Souza, Pedro Alejandro Gordan, Roberto Zonato Esteves, Izabel Cristina Meister Coelho

**Affiliations:** ^1^ Internal Medicine Department Health Sciences Center Londrina State University (UEL) Londrina PR Brazil; ^2^ Games Division Oniria Software Industry Londrina PR Brazil; ^3^ Medicine Department Maringá State University (UEM) Maringá PR Brazil; ^4^ Pró-Ensino na Saúde Pequeno Príncipe College Pequeno Príncipe Complex Curitiba PR Brazil

**Keywords:** diabetes mellitus, insulin, video games, medical education, continuing medical education, educational technology

## Abstract

**Background:**

Most patients with diabetes mellitus (DM) are followed by primary care physicians, who often lack knowledge or confidence to prescribe insulin properly. This contributes to clinical inertia and poor glycemic control. Effectiveness of traditional continuing medical education (CME) to solve that is limited, so new approaches are required. Electronic games are a good option, as they can be very effective and easily disseminated.

**Objective:**

The objective of our study was to assess applicability, user acceptance, and educational effectiveness of InsuOnline, an electronic serious game for medical education on insulin therapy for DM, compared with a traditional CME activity.

**Methods:**

Primary care physicians (PCPs) from South of Brazil were invited by phone or email to participate in an unblinded randomized controlled trial and randomly allocated to play the game InsuOnline, installed as an app in their own computers, at the time of their choice, with minimal or no external guidance, or to participate in a traditional CME session, composed by onsite lectures and cases discussion. Both interventions had the same content and duration (~4 h). Applicability was assessed by the number of subjects who completed the assigned intervention in each group. Insulin-prescribing competence (factual knowledge, problem-solving skills, and attitudes) was self-assessed through a questionnaire applied before, immediately after, and 3 months after the interventions. Acceptance of the intervention (satisfaction and perceived importance for clinical practice) was also assessed immediately after and 3 months after the interventions, respectively.

**Results:**

Subjects’ characteristics were similar between groups (mean age 38, 51.4% [69/134] male). In the game group, 69 of 88 (78%) completed the intervention, compared with 65 of 73 (89%) in the control group, with no difference in applicability. Percentage of right answers in the competence subscale, which was 52% at the baseline in both groups, significantly improved immediately after both interventions to 92% in the game group and to 85% in control (*P*<.001). After 3 months, it remained significantly higher than that at the baseline in both groups (80% in game, and 76% in control; *P*<.001). Absolute increase in competence score was better with the game (40%) than with traditional CME (34%; *P*=.01). Insulin-related attitudes were improved both after the game (significant improvement in 4 of 9 items) and after control activity (3 of 9). Both interventions were very well accepted, with most subjects rating them as “fun or pleasant,” “useful,” and “practice-changing.”

**Conclusions:**

The game InsuOnline was applicable, very well accepted, and highly effective for medical education on insulin therapy. In view of its flexibility and easy dissemination, it is a valid option for large-scale CME, potentially helping to reduce clinical inertia and to improve quality of care for DM patients.

**Trial Registration:**

Clinicaltrials.gov NCT001759953; https://clinicaltrials.gov/ct2/show/NCT01759953 (Archived by WebCite at http://www.webcitation.org/6oeHoTrBf)

## Introduction

Diabetes mellitus (DM) is a main public health problem of 21st century, affecting 422 million adults worldwide [1]. In most countries, most patients with DM are followed and treated by doctors who are not specialists in Endocrinology or diabetes, mainly primary care physicians (PCPs) [2]. However, only 24-56% of patients with DM present good glycemic control [3,4], defined as plasma levels of glycated hemoglobin A1c<7% [5].

Many factors may be implied in the low frequency of good metabolic control among patients with diabetes, but it is likely that one of the main reasons may be PCPs’ lack of knowledge and confidence on several aspects of DM management [6], specially regarding insulin use [7].

This gap in PCPs’ competence to treat diabetes with insulin contributes to the problem known as clinical inertia, “the failure to advance therapy when indicated,” [8] resulting in underuse of insulin [9] and poor glycemic control. In fact, previous reports show that there is an average delay of about 3-5 years between the first demonstrations that a patient with DM requires insulin and the actual initiation of insulin therapy [10,11].

Continuing medical education (CME) on DM and insulin is often advocated as a solution to optimize the knowledge and the practice of PCPs [12]; however, traditional CME activities (such as lectures and group discussions) have small and short-lasting efficacy [13]. Thus, new educational methods are urgently required. Electronic games are a powerful tool for education [14], as they “create a tight marriage among content, game play, and valued ways of thinking and acting” [15]. Reasons for using games include the familiarity of most college students with this medium [16] and their favorable views on the matter [17,18]. Nevertheless, the 2 most compelling arguments sustaining the adoption of games for learning are (1) their potential educational effectiveness [15,16] and (2) their flexibility and easy dissemination [19].

Good learning games are usually built following the same rules that guide the design of effective learning activities, which include stimulus to players’ intrinsic motivation, practice and repetition, effective feedback, arousal of positive feelings, intensity of the experience, and learner choice and involvement [20,21]. In the medical area, allowing students to practice their skills in a game may increase the safety for real patients [22].

Also, electronic games are learning resources much more flexible than traditional onsite educational activities, as they can be used in learners’ own equipment, location, and time schedule, rendering them more scalable [23].

In the field of diabetes, some games for education of patients [24-28] and a few technology-based initiatives for education of health professionals [13,29-34] have been described, but to our knowledge, no game has been previously reported for education of health professionals on diabetes or insulin.

We have previously described the process of design and development of InsuOnline, the first electronic serious game intended for medical education on insulin therapy for diabetes [35] and a formative assessment of usability and playability of its prototype [36].

A previous pilot study with medical students and residents has shown that InsuOnline was applicable and well accepted as a tool for medical education on insulin therapy, in what we called “real-world conditions” for self-directed distance learning activities: as a stand-alone resource, played in learners’ own equipment, in learners’ own time schedule, with minimal or no external guidance [36,37].

The next step in InsuOnline validation process is assessing its educational effectiveness.

Thus, the objectives of this study were to assess applicability, user acceptance, and effectiveness of InsuOnline for education of PCPs on insulin therapy for diabetes, as compared with a traditional onsite CME session with the same content and duration.

## Methods

### Trial Design

We performed an open-label randomized controlled trial to assess the effectiveness of InsuOnline as a method for education of PCPs on insulin therapy for diabetes, as compared with a “traditional” onsite educational activity with the same content and same duration.

### Eligibility Criteria

Subjects were eligible if they were medical doctors with an active register at a regional Council of Medicine in Brazil, were not specialists in Endocrinology or diabetes, were currently working at a public health care unit as a primary care physician (PCP), and were directly involved in the treatment of patients with diabetes in those facilities, with any degree of computer or gaming literacy.

### Recruitment

Researchers contacted local public health authorities of some cities from the states of Paraná (Londrina and other cities from the 17th Health Regional, Curitiba and São José dos Pinhais) and Santa Catarina (Blumenau), who agreed to help with recruitment. Those authorities have enlisted PCPs in their area to attend a training session about diabetes, and then we contacted those PCPs via phone, mail, or email to invite them to participate in the study.

Those who agreed to participate were informed of the research procedures and were sent by email a link to an online informed consent form stored on Google Drive (Multimedia Appendix 1). Respondents were allocated to groups by simple randomization using an online random number generator [38] by the first author. Due to our expectancy of a higher attrition rate in the game group, allocation was made at a 2:1 ratio.

### Settings and Locations Where Data Were Collected

After filling the informed consent form, participants were randomized and informed about the group they were allocated to and received an email with a link to the baseline questionnaire (stored on Google Drive). Also, participants allocated to control group were sent an email with information about the time and location of their scheduled onsite learning activity. A printed questionnaire was applied to those participants of the onsite learning activity immediately after its ending.

Participants allocated to game group received an email with instructions on how to download and install the game, after filling the baseline questionnaire, and then were contacted (weekly, if needed) to check if they have finished the game. After that, they were sent the link to another Web-based questionnaire.

After 3 months, a link to the third questionnaire was sent to participants of both groups.

If the participants did not answer the questionnaire after a few days, reminders were sent at 3-7–day intervals (initially by email, then by text message, and finally by phone). If they did not answer after up to 6 consecutive reminders, they were considered as loss of follow-up.

### Interventions

#### Game

**Figure 1:**
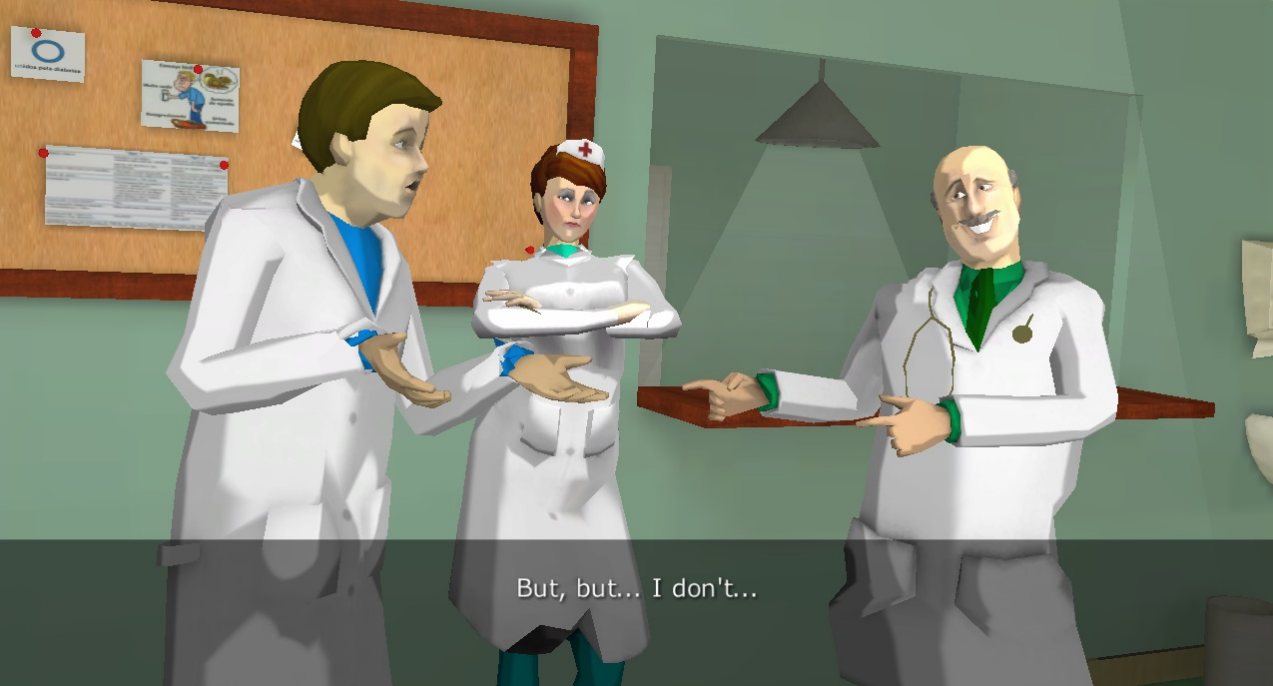
InsuOnline main characters: the young Dr Lucas (in the left) is the player’s avatar; the nurse Mariana (in the middle) gives useful hints; and the experienced Dr Braga (in the right) is a “mentor” who helps the player in critical moments.

**Figure 2:**
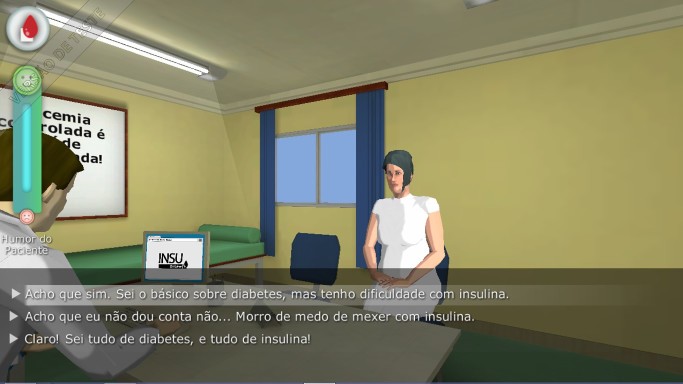
The player’s avatar, Dr Lucas, interacts with one of the patients in the game (dialogue in Portuguese).

InsuOnline was developed by transdisciplinary collaboration from a team composed by clinical endocrinologists, game designers, and experts in medical education, with the help of other professionals (programmers, graphic designers, sound editors, etc) when needed at specific points of the process, using the methodology of iterative prototyping [39]. The game was designed to be a tool for education of PCPs on how to best use insulin in the treatment of patients with DM, in a primary health care setting. Educational objectives of the game are presented in Textbox 1. InsuOnline was developed as a 3D app, with simple commands (all player actions were made using a mouse); game engine was built using Unity, and visual elements (scenarios, characters, animations) were designed on Blender 3D creation suite. A detailed description of InsuOnline design and development process can be found in [35]. The version used in this study was a code release (version 1.6.1); screenshots (in Portuguese) are shown in Figures 1 and 2.

Participants allocated to the game group received a personal login and password to access a website from where the game could be downloaded and installed in players’ own desktops or notebooks with Windows or MacOS, without any cost for study subjects.

During the game, players take on the role of a young doctor in a primary health care unit, whose mission is to improve glycemic control of an increasing-complexity series of 19 patients with diabetes, usually by means of initiating or adjusting insulin. Patients and situations presented in the game were designed to be similar to scenarios most commonly seen in a primary health care unit. The game gives immediate feedback after each player’s decision, and the player’s progression in the game is only possible if the most adequate decisions are made.

Several pedagogical elements were included in the game, aiming for the best educational effects [21,40]. These were based on the principles of adult learning and problem-based learning, including motivation, goal-orientation, relevancy-orientation, self-pacing, timely and appropriate feedback, contextualization, and practical (ie, hands-on) approach with active participation of the learner [41-43].

Recommendations about how to use insulin were selected from main clinical guidelines [5,44-49] and adapted to the reality of Brazilian public primary health care. Only the insulins commonly available in Brazilian public primary health care units (NPH and regular) were made available in the game. The software recorded players’ progress in the game. Participants were allowed to play the game in their preferred time and place, in the number of sessions they wanted. The amount of time necessary to finish the game was about 4 h in a previous study [37]. Usability, playability, and preliminary educational effectiveness of previous versions of the game were previously assessed and reported [36,37]; development was “frozen” during the trial.

Players were sent minimal written instructions on how to download and play the game, and most did so with minimal or no external guidance. If subjects had any difficulties, researchers were available to give remote assistance by email, text message, or phone, at any moment.

**Educational objectives of InsuOnline.**

- Recognize the goals of glycemic control in adults with DM
- Know when to start insulin in type 2 DM
- Know how to start insulin in type 2 DM (“bedtime” scheme)
- Know how to orientate proper insulin use (storing, injection technique, and devices)
- Know how to prevent, recognize, and treat hypoglycemia
- Know how to orientate and to interpret self-monitoring of blood glucose
- Know when and how to adjust insulin dosage
- Compare types of insulin (NPH, regular) and know when to use which
- Know when to intensify insulin therapy in type 2 DM
- Know how to intensify insulin therapy in type 2 DM (“basal-plus” scheme)
- Know how to prescribe intensive insulin therapy (“basal-bolus” scheme)
- Know how to recognize type 1 DM and how to start treatment
- Know how to recognize diabetic ketoacidosis and to start treatment
- Recognize the main barriers to insulin initiation and know how to address them
- Know how to orientate lifestyle changes and how to manage oral antidiabetic drugs in association with insulin
- Recognize the main factors that require insulin dosage adjustment and how to manage them

#### Onsite Learning Activity

Participants allocated to the “control” group (ie, the active comparator) were scheduled to join a traditional-format onsite learning session, comprising a series of 3 interactive expositive lectures and 2 group case discussions (the first with 3, and the second with 4 cases, which were identical to corresponding InsuOnline levels). PowerPoint slides for the lectures and printed case presentations were previously prepared by the main author, and their content was exactly identical to the recommendations included in the game. To avoid potential biases, lectures were given by a clinical endocrinologist not linked to the research team, previously trained by the main author and familiarized with the didactic material. Also, the onsite learning activity was designed to have the same duration of the game (about 4 h).

### Outcomes

Outcomes were self-assessed using written questionnaires which were applied to participants of both groups at 3 time points. The questionnaires were composed by different subscales at each time point: (1) at baseline, “competence” and “attitudes” subscales, plus demographic and professional data (for both groups); (2) immediately post intervention, the same “competence” and “attitudes” subscales for both groups, plus “game evaluation” subscale (for game group) or “onsite activity” subscale (for control group); (3) 3 months post intervention, “competence” and “attitudes” again, plus “importance for professional practice” subscale (for both groups).

The subscale of “competence” included 9 multiple-choice questions to assess factual knowledge about insulin and 11 clinical case vignettes with multiple-choice questions to assess problem-solving skills regarding insulin initiation and adjustment, which were created by our research team based on InsuOnline’s educational objectives (Textbox 1) and content. The subscale of “attitudes” included 9 Likert-type scale questions to assess attitudes and beliefs regarding diabetes and insulin, which were adapted from previous surveys [7,50].

The subscale of “game evaluation” had 16 Likert-type scale questions to assess playability, user satisfaction, and perceived educational utility of the game, with 1 additional free text item for additional comments from the participants, which were freely adapted from a questionnaire used to assess another game [51].

The subscale of “onsite activity evaluation” was composed by 10 Likert-type scale questions regarding methodology, user satisfaction, and perceived educational utility of the activity, and 1 free text item to collect additional comments, which were based on the ones from the “game evaluation” subscale, but adapted to an onsite CME session.

Finally, the subscale of “importance for professional practice” included 5 Likert-type scale questions and 2 free text items to assess participants’ opinion on the actual impact of the educational activity (game or onsite learning session) on their current professional practice, which were also created by authors.

All subscales were previously tested and refined in our pilot study with medical students and residents [36,37].

An additional subscale with 5 multiple-choice questions and 7 free text items was applied only to participants allocated to the game group who did not finish the game 60 days after receiving instructions for download or who did not respond to post-intervention questionnaires, to assess reasons why they were unable to play the game, and their perceptions about it.

All instruments used in this study are available in Multimedia Appendix 2.

Applicability of each intervention was defined as the proportion of participants allocated to each group that have received the intervention and did finish all tasks required in each intervention.

The primary outcome of this study was the score in the competence subscale in the immediately post-intervention self-assessment. Secondary outcomes were: score in competence subscale 3 months post-intervention; participants’ attitudes regarding diabetes and insulin post-intervention; participants’ acceptance and satisfaction with learning activities (game or onsite CME session); and applicability of the intervention (game or onsite CME session).

### Statistical Analysis

Only subjects who filled the baseline and the immediate post-intervention questionnaires were included in the analysis. Data from questionnaires were downloaded from Google Drive online forms or typed from printed questionnaires to worksheets on Excel (Microsoft Corporation) and then analyzed on Epi-Info 7 (Centers for Disease Control, Atlanta, Georgia) for descriptive analysis. Demographic data were compared among groups at baseline using chi-square test for proportions or Student *t* or Kruskal-Wallis test for continuous variables, as suited. Correlation among baseline scores on the competence subscale and demographic baseline data was assessed using Pearson *r* or Student *t* test on SPSS 14.0 (SPSS Inc). Comparison of the scores on competence subscale by group and by time point was performed by repeated-measures analysis of variance (ANOVA), with the Tukey post hoc test when a significant difference was detected, and by simple comparison between 2 groups at each time point using Student *t* test, on the statistical software R (The R Foundation). Proportion of subjects with scores of 90% or more on competence subscale and the frequency of “agree” answers on the Likert-type scale items for assessing attitudes were compared by group and by time point using chi-square test (followed by post hoc pairwise comparisons using Bonferroni correction when significant). Absolute increase in competence score from baseline to immediately post intervention was estimated on Epi-Info 7. The effect size of the group variable on the competence score was estimated using Cohen *d* test on R software. Reliability (internal consistency) of the subscales used in this study was evaluated using Cronbach alpha. Comments made by subjects in the free text items were reviewed by content analysis. Statistical significance was defined as *P*<.05.

#### Sample Size

In order to detect a minimum standard deviation of 0.5 on the score (percentage of right answers) in competence subscale, with 80% of statistical power at 5% of significance level, we estimated a sample size of 128 subjects (64 in each group). Taking into account an expected attrition rate of 40% in the game group (about twice the 17% attrition rate observed in the previous pilot study with students and residents [36,37]) and of 20% in the control group, we decided to enroll at least 90 subjects in the game group and 77 subjects in the control group.

### Ethics and Informed Consent

Participation was anonymous and voluntary, and all subjects filled an informed consent form (Multimedia Appendix 1), in accordance with Brazilian Health Ministry’s regulations for research on human beings. The study protocol was previously approved by Londrina State University Research Ethics Committee (UEL, #051/2011 and #051/2012), and by all local public health authorities from the cities where the study was performed.

## Results

### User Statistics

**Figure 3:**
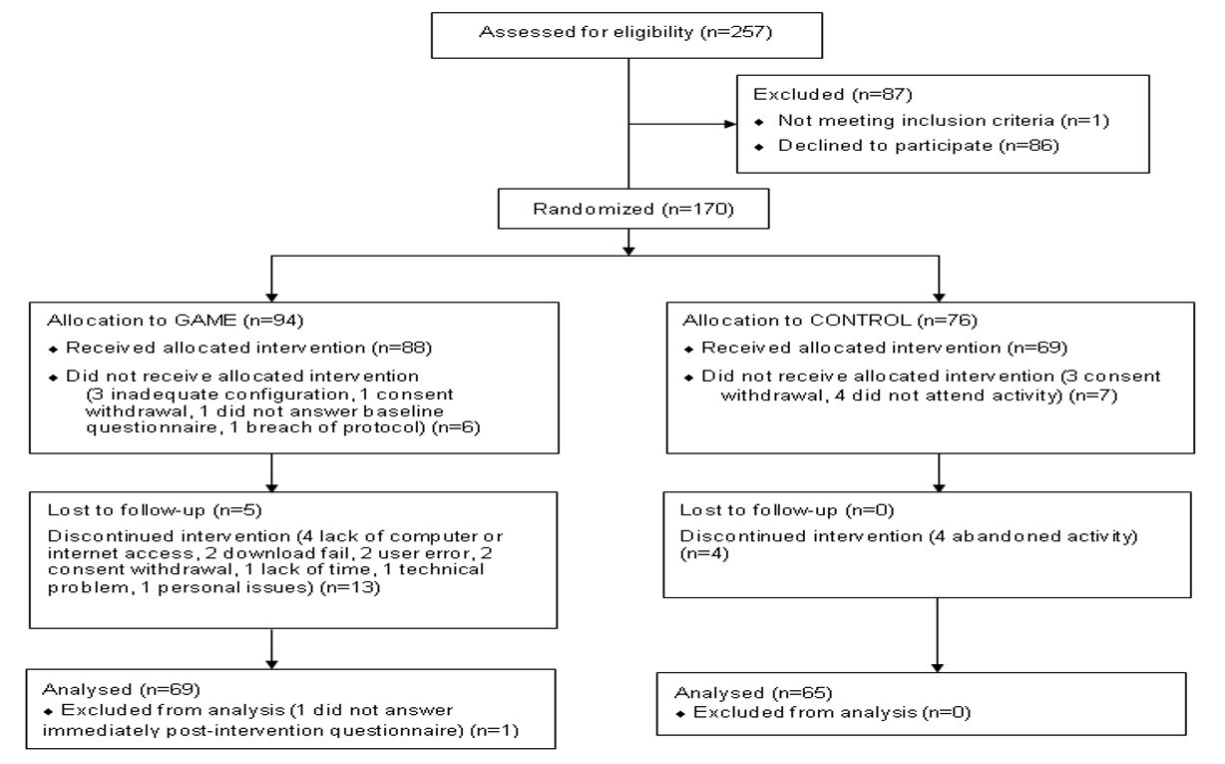
CONSORT flow diagram.

A total of 257 primary care physicians were contacted and assessed for eligibility during the recruitment period (July 2014 in Curitiba and São José dos Pinhais, Paraná; August 2014 in the cities from the 17th Health Regional of Paraná State except Londrina; September to October 2014 in Londrina, Paraná; and October 2014 in Blumenau, Santa Catarina); 170 were randomized, and 134 were included in the final analysis (however, 4 subjects from the game group and 7 from the control did not answer the third questionnaire, 3 months post-intervention). The CONSORT diagram [52] for participant flow is shown in Figure 3.

Baseline characteristics of subjects in both intervention groups were comparable, with no significant differences (Table 1).

**Table 1:** Characteristics of subjects allocated to each intervention group (game or control) at the baseline (n=134).

Characteristics of subjects		Game group (n=69)	Control group (n=65)
**Gender, n (%)**			
	Male	35 (50)	34 (52)
Age in years, mean (SD)		37.5 (11)	38.5 (12.7)
**Location, n (%)**			
	Curitiba	34 (49)	25 (38)
	17th HR^a^	20 (29)	19 (29)
	Londrina	15 (22)	18 (28)
	Blumenau	0	3 (5)
Years from graduation, median (range)		6.5 (0-41)	9.0 (0-41)
Years of experience in primary care, median (range)		4.0 (0-38)	5.0 (0-41)
Did residency, n (%)		27 (38)	25 (39)
**Residency area, n (%)**			
	Family and community health	8 (30)	9 (36)
	Internal medicine	8 (30)	4 (16)
	Ob&Gyn	4 (15)	2 (8)
	Surgery	4 (15)	4 (16)
	Pediatrics	2 (7)	3 (12)
	Other	1 (4)	3 (12)
**Self-referred number of patients seen per month with: median (range)**			
	type 2 DM	50 (4-300)	50 (1-500)
	type 2 DM on insulin	20 (0-150)	10 (1-120)
	type 1 DM	2 (0-100)	2 (0-80)

^a^17th HR: 17th Health Regional of Paraná state (group of 21 cities around Londrina, with the exception of Londrina).

### Outcomes

#### Applicability

Applicability, defined as the percentage of subjects who received the intervention and finished it, was 78% (69 of 88 subjects) for the game, and 89% (65 of 73 subjects) for the control onsite learning activity, with no significant difference between the two interventions (*P*=.11, chi-square test). Among the subjects who did not finish the game, 13 responded to our contact; reasons alleged for not finishing InsuOnline were mostly nonrelated to the game, such as lack of access to the Internet, lack of computer, or lack of time for playing it. Only 2 subjects were unable to access or download the game and 1 was unable to visualize it correctly in her computer due to incompatibility with other software (antivirus).

Among the game group subjects who did finish playing the game, the mean time required for finishing it after download instructions were sent was 21 days (SD 12, range 2-59). No difference was found in game applicability or in the time for finishing the game by age group, location, or gender of the participants.

#### Evaluation of the Interventions by Participants

Both interventions were very well rated by participants, as revealed by their responses to the subscales of “game evaluation” (in the game group) and “onsite activity evaluation” (in the control group), applied immediately after the interventions (most relevant data are summarized in Table 2).

Comparing similar questions among both groups, significantly more subjects have found the game “fun” (*P*=.04, chi-square test), without any other difference. The onsite activity was deemed “pleasant” by 83% of control subjects.

About two-thirds (68%) of game subjects strongly agreed that they have “learned more about insulin with this game than they would learn from a lecture,” and 68 of 69 (98%) would recommend the game for their friends.

In the free text field for comments, some subjects expressed their intense satisfaction with the game (“it is always better to learn by playing”), and even physicians who had never played an electronic game were able to play it and enjoyed the experience. Also, some criticisms were reported, related to game content (some repetitive or too extensive dialogues, soundtrack too loud) or to the software itself (some “freezes” when the player clicks too fast, incompatibility with some antivirus software).

Regarding the onsite activity, most comments were also highly positive (“excellent lecture,” “very practical approach,” “very dynamic and practical activity”). Criticisms were about the short duration of the activity (“too much information for one session only,” “it would be better addressed in two consecutive days”).

**Table 2:** Evaluation of interventions (game and onsite learning activity) by participants from each study group.

Items	Game group (n=69)		Control group (n=65)	
	Strongly agree, n (%)	Partially agree, n (%)	Strongly agree, n (%)	Partially agree, n (%)
The activity was fun	48 (70)^a^	18 (26)	32 (49)	21 (32)
The activity captured my attention all the time	43 (62)	25 (36)	47 (72)	15 (23)
I would join similar activities in the future	62 (90)	6 (9)	63 (97)	1 (2)
Patients presented in the activity were similar to the ones I see in my practice	43 (62)	22 (32)	46 (71)	16 (25)
The activity increased my knowledge about diabetes	66 (96)	3 (4)	60 (92)	5 (8)
The activity will influence the way I treat patients with diabetes	67 (97)	2 (3)	59 (91)	6 (9)

^a^*P*=.04 (χ^2^ test), compared to control group.

#### Competence for Prescribing Insulin

In relation to the primary outcome of this study, the percentage of right answers in the competence subscale (factual nowledge + problem-solving skills related to insulin) was about 52% at baseline in both groups, and it was significantly increased post-intervention (*P*<.001, ANOVA for repeated measures) in both groups. In the post hoc Tukey test, this increase was significant in the comparison between the baseline and immediate post-intervention time points (*P*<.001), between immediate post-intervention and 3 months post-intervention (*P*<.001), and between 3 months and the baseline (*P*<.001), which means that scores increased significantly in the immediate post-intervention time compared with baseline, then decreased significantly after 3 months in comparison with immediate post-test, but even with that decrease, 3-month scores remained significantly higher than at baseline.

**Figure 4:**
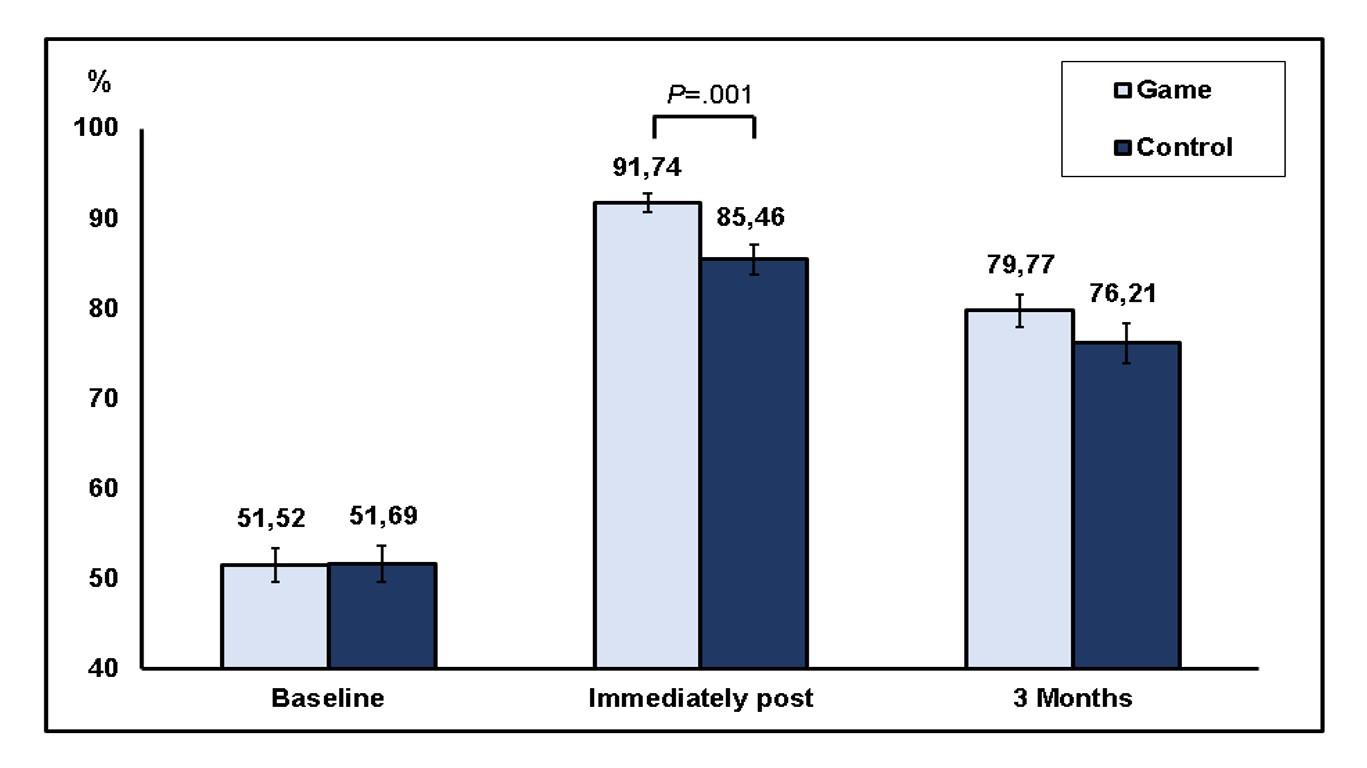
Score in competence subscale in both groups (game and control) in the 3 time points (baseline, immediately post-intervention, and 3 months post-intervention; mean and standard error).

When the effects of both factors “time” and “group” were taken into account, a significant difference was found related to “time” (*P*<.001) and to the iteration “group per time” (*P*=.02), but no difference was found related to the “group” isolatedly (*P*=.27, ANOVA for repeated measures). A simple comparison of competence score between the 2 groups at each time point showed no significant difference at baseline (51.5 [SD 15.6%] in the game vs 51.7 [SD 16.6%] in the control group; *P*=.95) and at 3 months (79.8 [SD 14.4%] in the game vs 76.2 [SD 16.9%] in the control group; *P*=.20), but a significantly higher score in the game group than in the control group at the immediate post-intervention assessment (respectively: 91.7 [SD 8.9%] vs 85.5 [SD 13.4%]; *P*=.001, Student *t* test; Figure 4).

In fact, absolute increase in competence score (from baseline to immediately post intervention) was higher in the game group (40 [SD 15%]) than in the control group (34 [SD 15%]; *P*=.01); that difference was marginal at 3 months (28 [SD 14] in the game vs 23 [SD 17] points in the control group; *P*=.06).

Also, the frequency of subjects who achieved a 90% or higher score in competence subscale at immediate post-intervention assessment was higher in the game group (53 subjects, or 77%) than in the control group (35 subjects, or 54%; *P*<.001), with no difference at baseline (0 in game vs 3% in the control group) or 3 months post-intervention (32% in game vs 26% in the control group).

Cohen *d* size effect of the game group compared with the control group was 0.4583 (0.093-0.8327) in the immediate post-intervention time [53].

No differences were observed in competence score by gender, location, residency status, years of experience in primary care, or self-reported mean number of patients with diabetes seen per month; a significant correlation was only observed with participants’ age (Pearson *r*=−.3314 at the baseline; *P*<.001; and Pearson *r*=−.4616 at immediate post-intervention time; *P*<.001).

#### Additional Outcomes

Significant improvement in diabetes and insulin-related attitudes from the baseline to post-intervention was observed in 4 of the 9 items in the game group, and in 3 of 9 items in the control. In post hoc analyses, most differences occurred between the baseline and immediate posttest, and a few between the baseline and 3 months. An additional statistical difference was observed between 3 months and immediate posttest in the control group in an item that assessed a personal opinion. Results for attitudes subscale are in Multimedia Appendix 3.

Three months after the interventions, about 80% of the subjects from both the groups stated that the activity had had real impact on their practice and that they knew better what to do when seeing a real patient with diabetes. About 62-75% of participants in both groups also said they were feeling more secure, and found it easier to help patients to improve their glycemic control. In the game group, significantly more subjects said that “it got easier” to orientate DM patients about their therapy, compared with control group (85% vs 66%; *P*=.03). Results from the subscale of “importance for professional practice” are presented in Multimedia Appendix 4.

Reliability of the subscales used in this study was estimated to be about 0.7-0.8 for most subscales (Cronbach alpha=.715 for competence subscale, .739 for game evaluation subscale, .649 for onsite activity evaluation, and .850 for importance for professional practice), except for attitudes subscale (Cronbach alpha=.323) [54].

No harms or adverse effects were reported.

## Discussion

### Principal Findings

Our results have shown that the game InsuOnline, used in real-world conditions (in players’ own computers and in their own flexible time schedule) was highly effective for education of PCPs on insulin therapy for diabetes, compared with an onsite CME activity with the same content and same approximate duration. In fact, competence improvement was better with the game than with the onsite learning session. InsuOnline was also applicable, with about 80% of the subjects with the more varied degrees of computer or gaming literacy being able to finish it with little or no external help. Both interventions were very well-rated by participants, regarding engagement, realism, and perceived educational value. The good evaluation of the onsite CME session proves that InsuOnline was compared with a valid control activity (a gold-standard active comparator). Three months after both the interventions, doctors from both the groups said that they were feeling more secure and more prepared to help real patients with diabetes in their daily professional practice, with even better results in the game group.

Games and simulators are being increasingly used for education of health care professionals on various topics [55-57], but, to our knowledge, this is the first report on the educational effectiveness of an electronic game for education of medical doctors about insulin therapy for diabetes.

Games are a promising way to deliver CME, for many reasons. One reason is their educational adequacy: well-designed games incorporate all the main principles of Adult Education, such as individualized, self-pacing, contextualized learning, with active experimentation and appreciation of previous knowledge [15,16,40-43]. The inclusion of game elements adds entertainment to the learning experience, increasing learners’ motivation to practice and to learn, which renders the learning experience more enjoyable, more engaging, and potentially more effective [40,58]. Games can be designed to simulate an infinite number of medical problems, and are easily updated as needed [15,40]. Finally, games are more flexible than traditional CME, respecting learners’ own time availability and learning pace, and are much more scalable [23]. So, we believe that well-designed and validated games will soon become the gold standard option for delivering large-scale CME.

Validation of games for health professional education is a recent and growing research area. Most available games for health professionals’ education have not passed through an objective validation process, and many of the published studies on the field are biased [59]. So, there is a great need of good quality studies which can provide solid evidence to support the fast-growing science of games for health. Our randomized-controlled trial was designed to contribute to this body of evidence by following the guidelines for research on health games effectiveness as proposed by Kato, who suggested that studies in this area should attempt to apply the same scientific rigor typical of health sciences [60].

As there is a variety of proposed methods for validating games for health, we opted to use multiple methods for validation of InsuOnline. One of the simplest validation approaches was proposed by Olsen [61], and includes only 3 aspects: usability, playability, and educational effectiveness. The first two aspects were very well rated for InsuOnline, as previously reported [36], and our current results support the third one. However, the best method to validate a game that was designed for CME is the approach to validation of CME activities described by Moore [62], that proposes a continuum of 7 levels: (1) applicability (user participation), (2) user satisfaction, (3) gain of factual and procedural knowledge (learning), (4) gain of competence, (5) improvement of learner’s performance, (6) improvement of patient health, and (7) improvement of community health. InsuOnline has already met the first 4 criteria. The following 3 steps (change in PCP’s professional performance and improvement of their patients’ glycemic control, and, hopefully, improvement of populational risk of DM complications) will be the focus of future research.

Attitudes and beliefs of PCPs in our sample were similar, at the baseline, to the ones of family doctors in United States [7] and in Arab-speaking countries in the Middle East [50], although the Brazilian doctors who participated in our study seem to be more often inclined to perceive the initiation of insulin therapy and the training of the patient with DM for using insulin as difficult and complicated tasks (78% and 54% of our subjects, respectively, compared with 66% and 42% in United States, and 48% and 25% in Middle East), which underscores the urgent need for effective educational interventions.

Reasons for the better results observed with the game than with the onsite learning activity, in this study, can be related to characteristics of the games that foster learning and attitude (and behavioral) changes [63]:

1. The inclusion of behavioral change procedures into the game, such as: goal setting, decision-making, problem solving, goals re-evaluation, social rewards; and

2. The use of a story (the game plot), and the inclusion of behavioral change concepts into that story. Players’ identification and empathy for the story protagonist may contribute to change players’ attitudes, reflecting the attitudinal change that occurs with the protagonist character in the game, in a process known as “modeling” [63].

Also, the higher emotional involvement of the learner with a game than with a traditional CME activity is another likely factor, as affection is a powerful determinant for changing attitudes and behavior [64]. Intrinsic motivation, a very effective learning catalyst, probably is one of the biggest advantages of games in relation to more traditional modalities of education [65]. Our game was designed to produce a high level of players’ engagement, by careful disposition of different game elements (realism, identification with context and characters, increasing-difficulty challenges, humor, clear objectives, immediate and intense feedback for each players’ action, progress monitoring, and rewards). With that, the majority of participants stated that the game was “fun” and that it captured their attention all the time.

### Limitations

Some limitations of our study should be pointed out. First, blinding of the participants (and researchers) was not feasible due to the nature of the interventions, and that may have affected some of our results, especially the subscales of evaluation of the activities, as many subjects may have found it exciting or unusual to play an electronic game to learn about a medical topic. However, we think that our primary outcome (competence for prescribing insulin) was not compromised, as it was objectively assessed by standardized multiple-choice questions.

Second, the instruments used in this study were not previously validated, because they were developed by our team to assess the specific outcomes we addressed in this study. The use of customized instruments is strongly recommended by Moreno-Ger [66], who argues that generic questionnaires are usually not useful for assessing games that can be very different in their objectives, target audiences, and needs. At our favor, we can say that our instruments were extensively reviewed by our team of experts in endocrinology and medical education, which warrants their face and content validity, and our subscales have shown acceptable reliability, as measured by their Cronbach alpha in the range of .7 to .8, with an exception of the attitudes subscale, which had very poor internal consistency [54]. The authors decided to maintain this subscale because it had questions similar to the ones used in previous surveys [7,50], allowing their comparison with our results.

In third place, this study shows only intermediate outcomes (PCPs’ knowledge, skills, attitudes, competence regarding insulin initiation, and adjustment for diabetes), but we don’t know yet if the game will have any impact on actual health professionals’ performance or on their patients’ measures or outcomes (“hard” outcomes), which we expect to assess in future studies.

Finally, although PCPs’ competence for prescribing insulin is essential, it is very likely that a simple intervention on the education of medical doctors may be insufficient to induce a significant change in the process of care delivered to patients with DM in primary care. A multifaceted intervention, aiming at several aspects of DM management, should probably be more effective to improve care and control of DM on a primary health care level, but it would demand conjugated efforts from government and society to improve issues such as availability of multiprofessional teams, availability of better insulins and oral drugs, availability of reagent strips for self-monitoring of blood glucose, better access to diabetes education, and others.

### Generalizability

We think our results can be generalized for most primary care physicians worldwide, as our sample included medical doctors from different geographic areas, different age groups, different levels of specialization and experience, and any degree of computer or gaming literacy. Also, our game was tested in what we believe to be the “real-world conditions” for the use of an electronic game for distance CME: in players’ own computers (with a wide range of possible equipment configurations), with very little or no external help from our team, and in their own spare time. Our results show that InsuOnline is a flexible, applicable and scalable option for large-scale CME on diabetes.

### Conclusions

In this “real-world conditions” randomized controlled trial, InsuOnline, the first electronic serious game designed for medical education on insulin therapy for diabetes, was applicable, very well accepted, and highly effective for education, with even better results for improving primary care physicians’ competence and attitudes related to diabetes and insulin than a gold-standard onsite CME activity with the same content and duration. For this reason, we believe InsuOnline is a valid tool for large-scale CME on DM, with advantages manifested in its easy Web dissemination, customizable content, and accordance with Adult Learning principles. We hope it can contribute to improve PCPs’ performance and optimize the quality of care offered to patients with DM in primary care, eventually improving patients’ glycemic control and reducing the risk of DM complications.

## Appendix

Multimedia Appendix 1 Informed consent form.

Multimedia Appendix 2 Instruments (questionnaires) used in this study.

Multimedia Appendix 3 Results from the "attitudes" subscale.

Multimedia Appendix 4 Results from the "importance for professional practice" subscale.

Multimedia Appendix 5 CONSORT eHealth checklist V1.6.1.

## Abbreviations

A1c: glycated hemoglobin A1c
CME: continuing medical education
DM: diabetes mellitus
PCP: primary care physician
RCT: randomized controlled trial
