# Supplementary material for: InsuOnline, an Electronic Game for Medical Education on Insulin Therapy: A Randomized Controlled Trial With Primary Care Physicians
Source: J Med Internet Res. 2017 Mar 9;19(3):e72. doi: 10.2196/jmir.6944 (PMC5364323; doi:10.2196/jmir.6944)
Supplement: Multimedia Appendix 1 [file jmir_v19i3e72_app1.pdf]

Londrina State University / Pequeno Príncipe College

Informed Consent Form

Research project title:
“Development and Evaluation of a Web-Based Serious Game for Education of Health Professionals on Insulin Therapy for Treating Diabetes Mellitus**”**

Dear Doctor:

We would like to invite you to join our research study “*Development and Evaluation of a Web-Based Serious Game for Education of Health Professionals on Insulin Therapy for Treating Diabetes Mellitus*”, performed by researchers from Londrina State University (Universidade Estadual de Londrina - UEL) and from Pequeno Príncipe College (Faculdades Pequeno Príncipe - FPP).

The objective of the study is to assess if an electronic game, used as a distance learning tool, can be as useful as traditional educational activities for education of medical doctors on treatment of patients with diabetes with insulin, in a Primary Health Care setting.

Your participation is very important. You would be randomly allocated to participate in a traditional onsite learning activity OR to play the electronic game.

Before and after your participation in one of those activities, you would have to respond a questionnaire to assess your knowledge and your oppinions on the use of insulin for treatment of diabetes, as well as to assess the activity you joined (onsite learning activity or game). A new questionnaire would be sent to you 3 months later, to assess if you retain what you learned from the onsite activity or from the game.

We would like to ensure you that your participation is completely voluntary, and that you can refuse to participate or even give up at any moment, without any onus or loss to yourself. We also warrant that your information would be used for the objectives of this study only, and that your personal data would be treated with the most absolute secrecy and confidenciality, in order to preserve your identity.

After data analysis, all evidence of your participation would be destroyed, as well as the records of the computer game that show your participation would be erased.

The expected benefits from this study are: to demonstrate that a computer game, used as a distance learning tool, can be an educational strategy as good as the traditional onsite

continuing medical education activities for education on practical aspects of how to use insulin for treating patients with diabetes. With that, we hope to contribute to optimize primary care physicians' competence for treating diabetes, then improving the quality of care given to patients with diabetes.

There are no significant risks associated with your participation in this study, besides the need of you spending some time of yours to participate in the proposed learning activity, and also to respond the questionnaires before and after the interventions.

We inform that you will not pay or be payed to participate in this study. We ensure, however, that any expenses decurrent from your participation in this research will be compensated, when they were specifically due to your participation on the study.

If you have any doubt or if you need any additional information, you may contact the main researcher: **Dr. Leandro Arthur Diehl**, phone **(xx) xxxx-xxxx** or **(xx) xxxx-xxxx**, e-mail: **insulina.pesquisa@gmail.com** , or you may seek the Committee of Ethics involving Research with Human Beings from Londrina State University, located at 60 Robert Koch Avenue, Londrina, PR, Brazil, or via phone: (xx) xxxx – xxxx.

Londrina/PR, _______________, 2014.

Dr. Leandro A. Diehl - Main Researcher

I, ____ ,
after having been informed about research procedures, do agree in participate voluntarily in the study described above.

Signature:________________________________ Date:____________________________

Your information for contact:

E-mail: ____

Phone(s): _______________________________ /__________________________________
