# Supplementary material for: InsuOnline, an Electronic Game for Medical Education on Insulin Therapy: A Randomized Controlled Trial With Primary Care Physicians
Source: J Med Internet Res. 2017 Mar 9;19(3):e72. doi: 10.2196/jmir.6944 (PMC5364323; doi:10.2196/jmir.6944)
Supplement: Multimedia Appendix 2 [file jmir_v19i3e72_app2.pdf]

Diehl LA et al. InsuOnline, an electronic game for medical education on insulin therapy: a randomized controlled trial with primary care physicians.

APPENDIX 2

INSTRUMENTS (QUESTIONNAIRES) USED IN THIS STUDY

APPENDIX 2.1

QUESTIONS ON SUBJECTS' DEMOGRAPHIC AND PROFESSIONAL DATA

1. Number of license at the Regional Medical Council	(Free text)
2. Gender	( ) Male<br>( ) Female
3. Age	(Free text)
4. How many patients with type 2 diabetes do you use to see per month (approximately)?	(Free text)
5. How many patients with type 2 diabetes on insulin do you use to see per month (approximately)?	(Free text)
6. How many patients with type 1 diabetes do you use to see per month (approximately)?	(Free text)
7. How many years do you have since your graduation in medical school?	(Free text)
8. How many years do you have of experience in primary health care?	(Free text)
9. Did you do medical residency?	( ) Yes<br>( ) No
10. If you did residency, in what area?	(Free text)

APPENDIX 2.2

COMPETENCE SUBSCALE

Insulin-related problem-solving skills	(11 multiple-choice questions)
**Text of the question**	**Choices**
1. A 55 years-old patient has type 2 DM for 8 years now. He is usually in good control, but in the last 6 months he has been showing worsening of his glycemic control (last A1c: 8.5%, fasting plasma glucose: 200mg/dL) and weight loss, despite continuous use of 2 oral drugs in maximal doses (metformin and glyburide). He denies any dietary or alcohol abuse, use of other medications, or infections. The best option to improve his glycemic control at this point would be:	( ) Start NPH insulin at bedtime, 10 units, and maintain both oral drugs [a]<br>( ) Start NPH insulin 0.2 units/kg at breakfast, and maintain both oral drugs<br>( ) Start NPH insulin 0.5 units/kg at bedtime, maintain metformin, and stop glyburide<br>( ) Add a third oral antidiabetic drug
2. A 50 years-old patient with 70kg has type 2 diabetes diagnosed 10 years ago. He currently takes metformin 850mg tid, and insulin in the following regimen: - At breakfast: 30 units of NPH + 8 units of regular; - Before supper: 10 units of NPH + 8 units of regular. The patient reports recurrent hypoglycemias in the middle of the night (about 3 AM) and moderately high levels of fasting plasma glucose. In other times of the day, his glucose levels are OK. The best option in this case would be:	( ) Stop metformin<br>( ) Reduce supper NPH insulin by half<br>( ) Stop supper NPH insulin<br>( ) Delay nighttime NPH insulin for application at bedtime [a]
3. A 60 years-old patient with 80kg has type 2 diabetes for more than 15 years now. He is currently in use of maximal-dose metformin,	( ) Maintain current medications and add NPH insulin, 10 units before lunch

plus NPH insulin: 20 units at breakfast, and 12 units at bedtime. His plasma glucose levels throughout the day, with the exception of after supper, when his glycemia is persistently between 200 and 300mg/dL. He denies hypoglycemias. The best option to optimize his glycemic control would be:	( ) Maintain breakfast NPH, and change nighttime NPH from bedtime to before supper<br>( ) Increase breakfast NPH dose, and maintain bedtime NPH<br>( ) Maintain current medications and add regular insulin, 4 units before supper [a]
4. A 40 years-old previously healthy patient seeks for medical evaluation complaining of marked tiredness, being thirsty and visual turvation in the last 3 weeks. He has lost 5kg during that period, despite eating even more than usual. He does not take any medication. Lab tests show fasting plasma glucose: 230mg/dL, and A1c: 10%. Indicate the best initial treament for this patient:	( ) Lifestyle changes only<br>( ) Metformin<br>( ) Metformin + glyburide<br>( ) Insulin [a]
5. A 45 years-old 100-kg patient has type 2 diabetes for ten years now. He has initiated NPH insulin 20 units at bedtime two months ago, because he was unable to maintain a good glycemic control with oral drugs only (metformin and glyburide in maximal doses). He comes today with new exams: fasting plasma glucose = 180mg/dL, and A1c = 8%. His self-monitoring of capillary blood glucose also shows several readings from 120 to 200mg/dL before breakfast, in the last 60 days. He did not have any hypoglycemias, and has good adherence. The best option to improve his	( ) Change the time of the NPH injection, from bedtime to breakfast<br>( ) Add a second injection of NPH before breakfast<br>( ) Increase the dose of bedtime NPH insulin [a]<br>( ) Stop all oral drugs and initiate an intensive insulin regimen

glycemic control, at this point, would be:	
6. A 15 years-old teenager seeks medical attention because she has been having excessive thirst and polyuria in the last 3 weeks, that worsened in the last 3 days, when she also started feeling ill, with abdominal pain and vomits. She lost 4kg during that period, despite normal appetite. During examination, she is prostrated, moderately dehydrated, afebrile, tachycardic (110bpm), tachypneic, and hypotensive (90/50mmHg). Her capillary glucose at that moment was 432mg/dL. The most adequate immediate action is:	( ) Start hydration with IV saline and collect blood for lab tests (gasometry, electrolytes, ketones) [a]<br>( ) Start hydration with IV saline and give IM regular insulin 15 units<br>( ) Start hydration with IV Ringer lactate and start IV infusion of regular insulin 6 units/hour<br>( ) Give SC regular insulin 6 units and IV sodium bicarbonate 50mEq in 1 hour
7. A 25 years-old patient with 60kg has type 1 diabetes diagnosed 15 years ago, and currently takes insulin as follows: - Before breakfast: 16 units of NPH + 8 units of regular; - Before lunch: 6 units of regular; - Before dinner: 8 units of regular; - At bedtime: 14 units of NPH. His last A1c was 7.4%. His weight is stable, he has no hypoglycemias, and his dietary habits are very regular. His self-monitored blood glucose levels are as follows: - Fasting: from 90 to 140; - Before lunch: from 80 to 130; - 2 hours post-lunch: from 140 to 190; - Before dinner: from 70 to 120; - Bedtime: from 180 to 250. The best choice to improve his glycemic control would be:	( ) Increase breakfast NPH to 20 units<br>( ) Increase lunch regular to 10 units<br>( ) Increase dinner regular to 12 units [a]<br>( ) Increase dinner regular to 16 units
8. A lean previously healthy 20 years-old male was diagnosed with diabetes today in a	( ) Start metformin e glyburide and follow-up at primary health care

primary health care facility, due to suggestive clinical symptoms and a fasting plasma glucose = 320mg/dL). He has a healthy appearance, is well hydrated, orientated, afebrile, normotensive, and breathing with no difficulties, with only a mild ketonuria. He does not take any medication and does not have other people with diabetes in his familiy. Choose the best immediate treatment:	level<br>( ) Start NPH insulin once daily at bedtime and follow-up at primary health care level<br>( ) Start NPH insulin once daily at bedtime and referral to endocrinologist<br>( ) Start insulin in an intensive regimen and referral to endocrinologist [a]
9. A 90-kg patient with type 2 diabetes needs intensification of his antidiabetic therapy because he is very symptomatic and still presents very high glucose levels during all times of the day, with A1c >9.5% despite taking 2 oral drugs + bedtime NPH insulin. The most practical way to start an intensive insulin regimen using only insulins available in primary care, with an initial dose of 0.5 units/kg/day, would be:	( ) NPH 15 units at breakfast, lunch, and dinner<br>( ) NPH 10 units + regular 5 units at breakfast, lunch, and dinner<br>( ) NPH 20 units + regular 10 units at breakfast; NPH 10 units + regular 5 units at dinner [a]<br>( ) NPH 10 units + regular 10 units at breakfast; regular 10 units at lunch; NPH 15 units at bedtime
10. A 40 years-old patient with type 2 diabetes for 12 years now is currently on a mix of NPH + regular insulin twice a day (at breakfast and at dinner) plus metformin. She enrolled at a gym for trying to lose weight and will start to do aerobic exercises of moderate intensity, 45 to 60 minutes per session, just after breakfast. The most adequate adjustment in her insulin therapy for this situation is:	( ) Reduce by 20% to 50% her breakfast regular insulin, in the days she will exercise [a]<br>( ) Withhold her breakfast regular insulin, in the days she will exercise<br>( ) Reduce by 25% her nighttime NPH insulin, in the nights before exercise<br>( ) Increase her ingestion of symple carbohydrates during breakfast, in

	the days she will exercise
11. A 48 years-old patient has 8 years of diabetes and is currently taking NPH 20 units at bedtime, metformin and gliclazide in maximal doses. Her latter lab tests show fasting plasma glucose: 102mg/dL, and A1c: 7.6%. Her fasting glucose measurements are all between 80 and 120mg/dL. Her weight is stable and she does not complain of hypogycemias. At this time, the next step to try to improve her glycemic control would be:	( ) Increase bedtime NPH to 24 units<br>( ) Add a second injection of NPH, 10 units before breakfast<br>( ) Add glyburide<br>( ) Request monitoring of capillary blood glucose in different times during the day [a]
**Insulin-related factual knowledge**	**(9 multiple-choice questions)**
**Text of the question**	**Choices**
12. Which of the following insulins has a cloudy ("milky") appearance?	( ) Regular<br>( ) NPH [a]<br>( ) Glargin<br>( ) All of them
13. NPH insulin, when injected in the subcutaneous tissue, presents PEAK and DURATION of action, respectively, in:	( ) 2 to 3 hours, and from 5 to 8 hours<br>( ) 3 to 4 hours, and from 8 to 12 hours<br>( ) 6 to 10 hours, and from 12 to 16 hours [a]<br>( ) 6 to 10 hours, and from 14 to 24 hours
14. Regarding insulin storing, preparation and injection techniques, all the following affirmations are correct, EXCEPT:	( ) NPH insulin must be rotated between hands 10 to 20 times before injection

	( ) Open insulin vials must be stored in the refrigerator exclusively [a]<br>( ) Insulin pen devices are easier to use for injection than syringes<br>( ) Shortest needles must be used for injection, even in obese patients
15. The glycemic goals for adults with diabetes, according with American Diabetes Association, are:	( ) fasting plasma glucose: 70 to 130; preprandial plasma glucose: 70 to 130; postprandial plasma glucose: under 180 [a]<br>( ) fasting plasma glucose: 70 to 100; preprandial plasma glucose: 100 to 140; postprandial plasma glucose: under 160<br>( ) fasting plasma glucose: under 100; preprandial plasma glucose: under 110; postprandial plasma glucose: under 140<br>( ) fasting plasma glucose: under 126; preprandial plasma glucose: under 140; postprandial plasma glucose: under 200
16. Type 2 diabetes is a progressive disease, in which patients continue to progressive lose their endogenous insulin secretion, until they eventually need exogenous insulin reposition to be able to obtain and maintain a good glycemic control. The percentage of patients with type 2 diabetes that will require	( ) 2% to 10%<br>( ) 10% to 25%<br>( ) 25% to 40%<br>( ) 40% to 80% [a]

exogenous insulin, after several years of disease, is estimated to be:	
17. All the following are signs of severe insulin deficiency:	( ) Involuntary weight loss, visual disturbances, gross glicosuria, hyperkalemia<br>( ) Involuntary weight loss, marked polyuria/polydipsia, plasma glucose >300, ketosis [a]<br>( ) Plasma glucose >250, pH <7,3, triglycerides >250, acanthosis nigricans<br>( ) Increased abdominal waist, high blood pressure, HDL <45, hyperuricemia
18. Please indicate which from the alternatives presents a correct choice of food for initial treatment of an episode of mild hypoglycemia:	( ) A chocolate bar<br>( ) A big glass of sugared milk<br>( ) A can of diet soda<br>( ) Half a can of regular soda [a]
19. Most common adverse effects of insulin therapy are:	( ) Allergy and worsening of diabetic neuropathy<br>( ) Weight gain and hypoglycemia [a]<br>( ) Lipoatrophy and hypokalemia<br>( ) Lipohypertrophy and increase of cardiovascular risk
20. The individualisation of glycemic goals in diabetes treatment is a current trend. Higher A1c goals (up to 8% or 8.5%) are preferrable in all the following situations, EXCEPT:	( ) Recent onset of diabetes [a]<br>( ) Fragile elderly patients<br>( ) Severe comorbidities with short life expectancy<br>( ) Severe kidney disfunction

[a] Correct answer.

APPENDIX 2.3

ATTITUDES SUBSCALE

Text of the question	Choices
I think it is best to delay insulin initiation until it is absolutely essential (after trying combination of several oral drugs in maximal doses)	( ) Strongly agree<br>( ) Partially agree<br>( ) Indifferent<br>( ) Partially disagree<br>( ) Strongly disagree
I think insulin therapy should be initiated by an endocrinologist, and not by a primary care physician	( ) Strongly agree<br>( ) Partially agree<br>( ) Indifferent<br>( ) Partially disagree<br>( ) Strongly disagree
I think insulin should be initiated into the hospital	( ) Strongly agree<br>( ) Partially agree<br>( ) Indifferent<br>( ) Partially disagree<br>( ) Strongly disagree
I think family physicians would prescribe insulin more often if it were not injectable	( ) Strongly agree<br>( ) Partially agree<br>( ) Indifferent<br>( ) Partially disagree<br>( ) Strongly disagree
I think insulin initiation is one of the most difficult aspects of treating patients with DM	( ) Strongly agree<br>( ) Partially agree<br>( ) Indifferent<br>( ) Partially disagree<br>( ) Strongly disagree
I think most patients with type 2 DM will eventually need insulin, regardless of their adherence to treatment	( ) Strongly agree<br>( ) Partially agree<br>( ) Indifferent<br>( ) Partially disagree<br>( ) Strongly disagree
I think that, for most patients, benefits of insulin therapy are greater than the risks	( ) Strongly agree<br>( ) Partially agree<br>( ) Indifferent<br>( ) Partially disagree<br>( ) Strongly disagree
I think that most patients would benefit from insulin therapy	( ) Strongly agree

before developing diabetes complications	( ) Partially agree<br>( ) Indifferent<br>( ) Partially disagree<br>( ) Strongly disagree
I think that, for most patients, training for using insulin is not complicated	( ) Strongly agree<br>( ) Partially agree<br>( ) Indifferent<br>( ) Partially disagree<br>( ) Strongly disagree

APPENDIX 2.4

GAME EVALUATION SUBSCALE

**Text of the question**	**Choices**
The game is fun to play	( ) Strongly agree<br>( ) Partially agree<br>( ) Indifferent<br>( ) Partially disagree<br>( ) Strongly disagree
The game captured my attention all the time	( ) Strongly agree<br>( ) Partially agree<br>( ) Indifferent<br>( ) Partially disagree<br>( ) Strongly disagree
I would like to try other similar games in the future	( ) Strongly agree<br>( ) Partially agree<br>( ) Indifferent<br>( ) Partially disagree<br>( ) Strongly disagree
Depth of topics presented in the game was appropriate	( ) Strongly agree<br>( ) Partially agree<br>( ) Indifferent<br>( ) Partially disagree<br>( ) Strongly disagree
Patients presented in the game were similar with the ones I usually see in my practice	( ) Strongly agree<br>( ) Partially agree<br>( ) Indifferent<br>( ) Partially disagree<br>( ) Strongly disagree
The game increased my knowledge about diabetes	( ) Strongly agree<br>( ) Partially agree<br>( ) Indifferent<br>( ) Partially disagree<br>( ) Strongly disagree
The game will influence the way I treat patients with diabetes	( ) Strongly agree<br>( ) Partially agree<br>( ) Indifferent<br>( ) Partially disagree<br>( ) Strongly disagree

The game was challenging	( ) Strongly agree<br>( ) Partially agree<br>( ) Indifferent<br>( ) Partially disagree<br>( ) Strongly disagree
Game instructions were enough for me to understand how to play it	( ) Strongly agree<br>( ) Partially agree<br>( ) Indifferent<br>( ) Partially disagree<br>( ) Strongly disagree
Tips given by the “mentor” during the game were useful for my learning	( ) Strongly agree<br>( ) Partially agree<br>( ) Indifferent<br>( ) Partially disagree<br>( ) Strongly disagree
Dialogue with the patients was useful for my learning	( ) Strongly agree<br>( ) Partially agree<br>( ) Indifferent<br>( ) Partially disagree<br>( ) Strongly disagree
Quizzes were useful for my learning	( ) Strongly agree<br>( ) Partially agree<br>( ) Indifferent<br>( ) Partially disagree<br>( ) Strongly disagree
The possibility of prescribing insulin and see what would be the result was useful for my learning	( ) Strongly agree<br>( ) Partially agree<br>( ) Indifferent<br>( ) Partially disagree<br>( ) Strongly disagree
I have learned more about insulin with this game than I would learn from a lecture	( ) Strongly agree<br>( ) Partially agree<br>( ) Indifferent<br>( ) Partially disagree<br>( ) Strongly disagree
I would recommend this game for my friends	( ) Strongly agree<br>( ) Partially agree<br>( ) Indifferent<br>( ) Partially disagree<br>( ) Strongly disagree
Do you have any additional comments on the game?	(Free text)

APPENDIX 2.5

ONSITE LEARNING ACTIVITY EVALUATION SUBSCALE

**Text of the question**	**Choices**
The activity was pleasant	( ) Strongly agree<br>( ) Partially agree<br>( ) Indifferent<br>( ) Partially disagree<br>( ) Strongly disagree
The activity was fun	( ) Strongly agree<br>( ) Partially agree<br>( ) Indifferent<br>( ) Partially disagree<br>( ) Strongly disagree
The activity captured my attention all the time	( ) Strongly agree<br>( ) Partially agree<br>( ) Indifferent<br>( ) Partially disagree<br>( ) Strongly disagree
I would join other similar activities in the future	( ) Strongly agree<br>( ) Partially agree<br>( ) Indifferent<br>( ) Partially disagree<br>( ) Strongly disagree
The way information was presented in the activity was appropriate	( ) Strongly agree<br>( ) Partially agree<br>( ) Indifferent<br>( ) Partially disagree<br>( ) Strongly disagree
Patients presented in the activity were similar with the ones I usually see in my practice	( ) Strongly agree<br>( ) Partially agree<br>( ) Indifferent<br>( ) Partially disagree<br>( ) Strongly disagree
The activity has approached most situations I usually see in my practice	( ) Strongly agree<br>( ) Partially agree<br>( ) Indifferent<br>( ) Partially disagree<br>( ) Strongly disagree

The activity increased my knowledge about diabetes	( ) Strongly agree<br>( ) Partially agree<br>( ) Indifferent<br>( ) Partially disagree<br>( ) Strongly disagree
The activity has increased my security for prescribing insulin to patients with diabetes	( ) Strongly agree<br>( ) Partially agree<br>( ) Indifferent<br>( ) Partially disagree<br>( ) Strongly disagree
The activity will influence the way I treat patients with diabetes	( ) Strongly agree<br>( ) Partially agree<br>( ) Indifferent<br>( ) Partially disagree<br>( ) Strongly disagree
Do you have any additional comments on the activity?	(Free text)

APPENDIX 2.6

IMPORTANCE FOR PROFESSIONAL PRACTICE SUBSCALE

**Text of the question**	**Choices**
The activity had impact on my actual practice	( ) Strongly agree<br>( ) Partially agree<br>( ) Indifferent<br>( ) Partially disagree<br>( ) Strongly disagree
After the activity, I felt I knew better what to do when seeing a patient with diabetes	( ) Strongly agree<br>( ) Partially agree<br>( ) Indifferent<br>( ) Partially disagree<br>( ) Strongly disagree
After the activity, I really felt more secure when seeing a patient with diabetes	( ) Strongly agree<br>( ) Partially agree<br>( ) Indifferent<br>( ) Partially disagree<br>( ) Strongly disagree
For me, it got easier to manage treatment of patients with diabetes	( ) Strongly agree<br>( ) Partially agree<br>( ) Indifferent<br>( ) Partially disagree<br>( ) Strongly disagree
I was able to help my patients with diabetes to improve their control, thanks to what I have learned in the activity	( ) Strongly agree<br>( ) Partially agree<br>( ) Indifferent<br>( ) Partially disagree<br>( ) Strongly disagree
If you did not succeed applying what you learned into your actual practice, please tell us why:	(Free text)
Additional comments:	(Free text)

APPENDIX 2.7

QUESTIONS SENT FOR SUBJECTS WHO DID NOT FINISH THE GAME OR DID NOT ANSWER POST-GAME QUESTIONNAIRES ("INSUCESS" SUBSCALE)

**Text of the question**	**Choices**
Did you try to download and install the game?	( ) Yes<br>( ) No
If you did NOT try to download and install the game, please tell us why:	(Free text)
Did you succeed downloading and installing the game in your computer?	( ) Yes<br>( ) No
If you did NOT succeed downloading and installing the game, please tell us why:	(Free text)
Did you begin to play the game?	( ) Yes<br>( ) No
If you did NOT begin playing the game, please tell us why:	(Free text)
If you DID begin playing the game, did you play up to what patient/level?	(Free text)
Please tell us what was the main reason you did NOT finish the game:	(Free text)
Was there any other reasons, despite the main reason indicated above, for you having NOT finished the game? What is/were that/those reason/reasons?	(Free text)
In your oppinion, what could be improved in the game that would help you to finish it?	(Free text)
Did you find the game fun to play?	( ) Yes<br>( ) No<br>( ) I did not play it
Did you find the game potentially useful for education on insulin?	( ) Yes<br>( ) No<br>( ) I did not play it
