# Supplementary material for: InsuOnline, an Electronic Game for Medical Education on Insulin Therapy: A Randomized Controlled Trial With Primary Care Physicians
Source: J Med Internet Res. 2017 Mar 9;19(3):e72. doi: 10.2196/jmir.6944 (PMC5364323; doi:10.2196/jmir.6944)
Supplement: Multimedia Appendix 3 [file jmir_v19i3e72_app3.pdf]

Diehl LA et al. InsuOnline, an electronic game for medical education on insulin therapy: a randomized controlled trial with primary care physicians.

APPENDIX 3

RESULTS FROM THE "ATTITUDES" SUBSCALE

Table 4. Attitudes related to diabetes and insulin in both groups (game and control) at baseline, immediately post-intervention, and 3 months post-intervention (frequency of "strongly agree" + "partly agree" responses).

	**Baseline**	**Immediately post**	**3 months post** [a]
	**Agreement (%)**	**Agreement (%)**	**Agreement (%)**
**I think it is best to delay insulin initiation until it is absolutely essential**			
Game group	43 (62%)	27 (39%) [b]	26 (40%) [b]
Control group	47 (72%)	28 (43%) [b]	28 (48%)
**I think insulin therapy should be initiated by an endocrinologist**			
Game group	9 (13%)	3 (4%)	1 (2%) [b]
Control group	13 (20%)	2 (3%) [b]	4 (7%)
**I think insulin therapy should be initiated in hospital**			
Game group	3 (4%)	1 (1%)	1 (1%)
Control group	4 (6%)	3 (5%)	5 (9%)

**I think family physicians would prescribe insulin more often if it were not injectable**			
Game group	43 (62%)	43 (62%)	45 (69%)
Control group	38 (58%)	35 (54%)	47 (81%) [c]
**I think insulin initiation is one of the most difficult aspects of treating patients with DM**			
Game group	56 (81%)	46 (67%)	53 (82%)
Control group	48 (74%)	50 (77%)	48 (83%)
**I think most patients with type 2 DM will eventually need insulin, regardless of their adherence**			
Game group	36 (52%)	65 (94%) [b]	57 (88%) [b]
Control group	32 (49%)	55 (85%) [b]	46 (80%) [b]
**I think that, for most patients, benefits of insulin are greater than the risks**			
Game group	60 (87%)	68 (99%)	62 (96%)
Control group	55 (85%)	64 (99%)	56 (97%)
**I think most patients would benefit from**			

**insulin before developing diabetes complications**			
Game group	64 (93%)	67 (97%)	61 (94%)
Control group	59 (91%)	63 (97%)	56 (97%)
**I think that, for most patients, training for using insulin is not complicated**			
Game group	31 (45%)	54 (78%) [b, d]	45 (69%)
Control group	30 (46%)	34 (52%)	30 (52%)

[a] 4 subjects from game group and 7 from control group did not answer the 3-month post-intervention questionnaire.

[b] *P*<.05 compared to baseline (pairwise chi-squared test with Bonferroni correction).

[c] *P*<.05 compared to immediately post-test (pairwise chi-squared test with Bonferroni correction).

[d] *P*<.05 compared to control group at the same point (chi-squared test).
