# Supplementary material for: InsuOnline, an Electronic Game for Medical Education on Insulin Therapy: A Randomized Controlled Trial With Primary Care Physicians
Source: J Med Internet Res. 2017 Mar 9;19(3):e72. doi: 10.2196/jmir.6944 (PMC5364323; doi:10.2196/jmir.6944)
Supplement: Multimedia Appendix 4 [file jmir_v19i3e72_app4.pdf]

Diehl LA et al. InsuOnline, an electronic game for medical education on insulin therapy: a randomized controlled trial with primary care physicians.

APPENDIX 4

RESULTS FROM THE "IMPORTANCE FOR PROFESSIONAL PRACTICE" SUBSCALE

Table 5. Opinions of the participants on the importance of the intervention (game or onsite activity) for their professional practice (frequency of "strongly agree" responses).

	**Game Group**	**Control Group**
	**n = 65** [a]	**n = 58** [a]
The activity had impact on my practice	55 (85%)	44 (76%)
After the activity, I felt I knew better what to do	52 (80%)	46 (79%)
After the activity, I really felt more secure when seeing a patient with DM	46 (71%)	36 (62%)
For me, it got easier to manage treatment of patients with DM	55 (85%)	38 (66%) [b]
I was able to help my patients to improve control with what I learned	49 (75%)	36 (62%) [c]

[a] 4 subjects from game group and 7 from control group did not answer the 3-month post-intervention questionnaire.

[b] P=.03 in the comparison between the groups (chi-squared test).

c P=.06 in the comparison between the groups (chi-squared test).
